# Exploring the implicit meanings of ‘cultural diversity’: a critical conceptual analysis of commonly used approaches in medical education

**DOI:** 10.1007/s10459-024-10371-x

**Published:** 2024-09-14

**Authors:** Albertine Zanting, Janneke M. Frambach, Agnes Meershoek, Anja Krumeich

**Affiliations:** 1 Department of Health, Ethics and Society, School of Health Professions Education, Faculty of Health, Medicine and Life Sciences, Maastricht University, Maastricht, The Netherlands; 2 School of Health Professions Education, Faculty of Health, Medicine and Life Sciences, Maastricht University, Maastricht, The Netherlands; 3 Department of Health, Ethics and Society, Faculty of Health, Medicine and Life Sciences, Maastricht University, Maastricht, The Netherlands

**Keywords:** Cultural diversity, Cultural competence, Intersectionality, Cultural humility, Critical consciousness, Critical review

## Abstract

Existing approaches to cultural diversity in medical education may be implicitly based on different conceptualisations of culture. Research has demonstrated that such interpretations matter to practices and people concerned. We therefore sought to identify the different conceptualisations espoused by these approaches and investigated their implications for education. We critically reviewed 52 articles from eight top medical education journals and subjected these to a conceptual analysis. Via open coding, we looked for references to approaches, their objectives, implicit notions of culture, and to implementation practices. We iteratively developed themes from the collected findings. We identified several approaches to cultural diversity teaching that used four different ways to conceptualise cultural diversity: culture as ‘fixed patient characteristic’, as ‘multiple fixed characteristics’, as ‘dynamic outcome impacting social interactions’, and as ‘power dynamics’. We discussed the assumptions underlying these different notions, and reflected upon limitations and implications for educational practice. The notion of ‘cultural diversity’ challenges learners’ communication skills, touches upon inherent inequalities and impacts how the field constructs knowledge. This study adds insights into how inherent inequalities in biomedical knowledge construction are rooted in methodological, ontological, and epistemological principles. Although these insights carry laborious implications for educational implementation, educators can learn from first initiatives, such as: standardly include information on patients’ multiple identities and lived experiences in case descriptions, stimulate more reflection on teachers’ and students’ own values and hierarchical position, acknowledge Western epistemological hegemony, explicitly include literature from diverse sources, and monitor diversity-integrated topics in the curriculum.

## Introduction

To prepare medical students for encounters with an increasingly diverse patient population, medical education aims to enhance their awareness of cultural differences, teach them how to engage with these differences, and how to become more competent doctors (Ang et al., 2020; Brottman et al., 2020; Dao et al., 2017; Dogra et al., 2010; Leyerzapf & Abma, 2017; Nazar et al., 2015; Shepherd, 2019; Worden & Ait-Daoud Tiouririne, 2018). Such education may adopt different ways of approaching cultural diversity and be underpinned by equally dissimilar theories and concepts, such as intercultural competence, cultural safety, intercultural sensitivity, and cultural consciousness (Ang et al., 2020; Brottman et al., 2020; Dao et al., 2017; Guerra & Kurtz, 2017; Jowsey, 2019; Leyerzapf & Abma, 2017; Nazar et al., 2015; Pimentel et al., 2021; Shepherd, 2019; Worden & Ait-Daoud Tiouririne, 2018). Previous studies have noted that the implementation of these theoretical approaches in educational practice is not always clear and easy, and could even have opposite effects such as polarisation and stigmatisation (Dao et al., 2017; Guerra & Kurtz, 2017; Leyerzapf & Abma, 2017; Nazar et al., 2015; Shepherd, 2019; Worden & Ait-Daoud Tiouririne, 2018), thereby detrimentally affecting educational quality and learning outcomes.

Although many of these approaches start from the assumption that culture matters in communication, education, and skills development, few seem to define ‘culture’ explicitly. Instead, they tacitly assume its meaning is familiar, implicitly conceptualising it in a certain way nonetheless. Moreover, studies in medical education have revealed that different interpretations of ‘culture’ exist (Gregg & Saha, 2006; Pratt & Schrewe, 2016). With this study, we aimed to render these conceptualisations explicit. This is crucial, because research has shown that it matters to the people and practices concerned which notion of culture is adopted. More specifically, studies outside medical education have revealed that the notion of cultural diversity—consciously or unconsciously employed—influences choices and policies in practice with major implications for those involved (Bowker & Star, 2008; Meershoek & Krumeich, 2009; Proctor et al., 2011; Yanow & van der Haar, 2013). Underlying epistemological and ontological choices seem to have important consequences. Van Dijk (1989), for instance, noted that care providers’ inaccurate perceptions of migrants’ culture as a duplicate of their home country’s village culture provided them with unhelpful tools, causing them to unconsciously use ‘culture’ as an alibi for failed care. Similarly, Shim (2005) demonstrated how epidemiologists’ understanding of race differed from how lay people living with cardiovascular disease tended to construct race in structural terms. She argued that the lived experiences of race undermined the validity of scientific measurement, in turn impacting health outcomes (Shim, 2005). Finally, in our previous study we found that culture was unconsciously constructed in Dutch course materials as something or someone exotic, deviant from a standard Dutch or Western patient or disease, and therefore problematic (Zanting et al., 2020). We argued that this embedded notion of ‘problematic stranger’ could deprive doctors of the appropriate tools to take medical action and feed insecurity among them.

These findings beg the question of how culture is conceptualised and employed in common approaches to cultural diversity education in medical education. We therefore reviewed medical education literature and performed a conceptual analysis, addressing the following research questions: What approaches to cultural diversity are commonly used in medical education? How is cultural diversity conceptualised in these approaches? And what are the implications of these conceptualisations? This study aimed to offer curriculum designers, educators and researchers insights into the merits and limitations of the approaches currently used and their consequences for cultural diversity education and how doctors engage with a diverse patient population.

## Methods

We performed a critical review of medical education literature (Grant & Booth, 2009), employing a conceptual analysis of ‘cultural diversity’ models. The study is designed within a critical theory paradigm, which aims to explore underlying power structures and is underpinned by core values of democracy and egalitarianism (Paradis et al., 2020). We used a broad definition of ‘model’ that includes concepts, models, theories, and frameworks. First, we purposefully searched for articles that were recently published (January 1, 2015–December 31, 2022) in the seven top medical education journals. In doing so, we used search terms that are commonly employed in the literature and in educational practice to describe how culture plays a role in communication, education, skills development, and cooperation. These search terms were: intercultural communication, intercultural competence, intercultural sensitivity, cultural consciousness, cultural diversity/difference, intercultural education, cultural competence, multicultural competence, intercultural maturity, cross-cultural adaptation, cultural intelligence, transcultural communication, cross-cultural awareness, cultural transition, critical consciousness, intersectionality, cultural humility, insurgent multiculturalism, cultural proficiency, cultural sensitivity, and cultural responsiveness.

Next, we screened titles and abstracts for articles that described an approach to engaging with cultural diversity in medical education. We included studies that focused on curriculum contents and design (learning/teaching how to treat culturally diverse patients) as well as studies that addressed student and staff interactions. We excluded articles that focused solely on the clinical context or addressed the organisational, feedback or assessment culture, the appraisal and admission of cultural minorities, or inter-country comparative studies. We scanned the reference lists of the selected articles and found that one article from an additional journal was often referred to and was explicitly described as an approach frequently used and we therefore added it to the original selection of seven journals. Consequently, the final selection included 52 articles from the following eight journals:

1. Academic Medicine: 14
2. Medical Education: 6
3. Medical Teacher: 7
4. Advances in Health Sciences Education: 5
5. Teaching and Learning in Medicine: 4
6. BMC Medical Education: 11
7. Perspectives on medical education: 4
8. Journal of Transcultural Nursing: 1

Third, we performed a conceptual analysis of the selected articles in an iterative process with the aim to ‘clarify meanings and boundaries of concepts to promote understanding and support future research’ (Kahn & Zeidler, 2017). All authors joined in reading the articles closely, although one author (AZ) reviewed the majority. Via open coding we looked for references to concepts and models, their objectives, implicit or explicit notions of culture, and to implementation practices and collected these in a chart. We then analysed this chart, compared the data and their relevance, and developed overarching patterns to answer the research questions.

### Reflexivity

To warrant trustworthiness, we consciously questioned our positionality within the research team and towards the data (Dodgson, 2019). We structured our reflections and discussed our interpretations of the data, which were shaped by our previous study (Zanting et al., 2020), our backgrounds as social scientists using constructivist approaches to research from a critical perspective, and our experiences and observations of cultural diversity education as teachers in medical and health professions programmes. We are employed by an institute that has a dominant publication record in the field of health professions education. In many situations and contexts, we have a privileged background in terms of being associated with this institute, our socio-economic status, and our ethnicity, for example. We share lived experiences of cultural diversity in our professional and personal lives, and continuously try to be aware of the fact that our backgrounds shape our views. However, we realize this awareness has its limitations due to an absence of lived experiences as persons not having this privilege. The reflexivity practiced during our discussions and the critical theory paradigm helped construct the results based on the data.

## Results

We identified several variably elaborated approaches that were presented as a concept, model, or framework about how to engage with diversity (cultural or otherwise) in medical education (see overview approaches and related articles in Table 1).

**Table 1:** Cultural diversity approaches identified in the articles included in the critical review on the meanings of cultural diversity (2015–2022), and their link to the four identified notions of cultural diversity

Cultural diversity approaches	Articles	Notion of cultural diversity
Biocultural anthropologic framework (BAF)	Clementz et al. (2017)	Notion 4: culture as power dynamics
Critical consciousness	Dao et al. (2017), Manca et al. (2020), Herzog et al. (2021), Zaidi et al. (2017), Zaidi et al. (2016), Cavanagh et al. (2019), Halman et al. (2017), Bleakley (2017)	Notion 3: culture as dynamic outcome impacting social interactions
		Notion 4: culture as power dynamics
Cultural awareness	Shepherd (2019), Worden and Ait-Daoud Tiouririne (2018), Liu and Li (2022)	Notion 1: culture as a fixed patient characteristic
		Notion 2: culture as multiple fixed characteristics
		Notion 3: culture as dynamic outcome impacting social interaction
Cultural/intercultural competence	Brottman et al. (2020), Leyerzapf and Abma (2017), Nazar et al. (2015), Shepherd (2019), Worden and Ait-Daoud Tiouririne (2018), Jowsey (2019), Kor et al. (2022), Liu and Li (2022), Jie et al. (2022), Uzelli Yilmaz et al. (2022), Klenner et al. (2022), Sawatsky et al. (2017), Le et al. (2022), Sorensen et al. (2019), Rothlind et al. (2021), Campinha-Bacote (2002), Hordijk et al. (2019), Matthews and Van Wyk (2018), Sklar (2018), Pang et al. (2021), Vasquez Guzman et al. (2021), Martinez et al. (2015), Krishnan et al. (2019)	Notion 1: culture as a fixed patient characteristic
		Notion 2: culture as multiple fixed characteristics
		Notion 3: culture as dynamic outcome impacting social interaction
		Notion 4: culture as power dynamics
Cultural hegemony	Kuper et al. (2016)	Notion 3: culture as dynamic outcome impacting social interaction
		Notion 4: culture as power dynamics
Cultural humility	Nazar et al. (2015), Shepherd (2019), Worden and Ait-Daoud Tiouririne (2018), Jowsey (2019), Matthews and Van Wyk (2018), Pang et al. (2021), Solchanyk et al. (2021), Martinez et al. (2015)	Notion 3: culture as dynamic outcome impacting social interaction
		Notion 4: culture as power dynamics
Cultural safety	Shepherd (2019), Guerra and Kurtz (2017), Jowsey (2019), Pimentel et al. (2021), Jie et al. (2022), Klenner et al. (2022), Kuper et al. (2016)	Notion 1: culture as a fixed patient characteristic
		Notion 3: culture as dynamic outcome impacting social interaction
		Notion 4: culture as power dynamics
Diversity, Equity, Inclusivity (DEI or EDI)	Uzelli Yilmaz et al. (2022), Holdren et al. (2022), Sukhera et al. (2022), Rossi et al. (2022)	Notion 2: culture as multiple fixed characteristics
		Notion 4: culture as power dynamics
Diversity education	Dogra et al. (2016)	Notion 3: culture as dynamic outcome impacting social interaction
		Notion 4: culture as power dynamics
Foucauldian perspective	Zaidi et al., (2021a, 2021b)	Notion 4: culture as power dynamics
Intersectionality	Uzelli Yilmaz et al. (2022), Bochatay et al. (2022), Blackie et al. (2019), Eckstrand et al. (2016), Muntinga et al. (2016), Peek et al. (2020), Sukhera et al. (2022)	Notion 2: culture as multiple fixed characteristics
		Notion 4: culture as power dynamics
Race consciousness	Sharma and Kuper (2017)	Notion 4: culture as power dynamics
Social accountability	Gruner et al. (2022), Fung and Ying (2022), Goez et al. (2020)	Notion 1: culture as a fixed patient characteristic
		Notion 3: culture as dynamic outcome impacting social interaction
		Notion 4: culture as power dynamics
Structural competence	Krishnan et al. (2019)	Notion 3: culture as dynamic outcome impacting social interaction

The approaches’ objectives were diverse and included addressing health care disparities, meeting special needs of specific patient groups, enhancing doctor-patient interaction, and reflecting and acting upon the power structures and inequities inherent in educational and health care processes. From our analysis, we concluded that the approaches conceptualised cultural diversity in four different ways. In the following paragraphs, we will describe each of these four notions, including their implications for education and limitations as pointed out in the analysed articles. Whereas we constructed each notion from several of the identified approaches listed in Table 1, some approaches also shared similarities with multiple notions. Notwithstanding this, we were able to link one prominent approach to each notion and presented these in Boxes 1–4. The third column in Table 1 shows the relation between the notions and the cultural diversity approaches.

**Box 1:** Description of ‘Cultural competence’ approach, identified in the articles included in the critical review on the meanings of cultural diversity (2015–2022)

**Cultural competence**: ‘involves people treating people in a way that makes them feel that their ideas, values, traditions, or behaviours are acknowledged and respected’ (Jowsey, 2019). It aims ‘to care for patients of different cultural, ethnic and social background’ (Hordijk et al., 2019), ‘to improve patient–provider communication by teaching medical students and physicians to better understand their patients’ race and culture’ (Krishnan et al., 2019), and ‘to teach a list of useful facts and general knowledge about different cultural groups so as to better understand patients’ (Nazar et al., 2015)	

### Culture as a fixed patient characteristic

In several approaches, cultural diversity was conceptualised as a difference between patients in terms of one particular characteristic, that is, their culture. As such, culture implicitly referred to people who spoke languages other than the national language or who had a different nationality (Kor et al., 2022; Nazar et al., 2015) or religion (Jie et al., 2022; Liu & Li, 2022; Uzelli Yilmaz et al., 2022), to indigenous or Aboriginal populations (Guerra & Kurtz, 2017; Klenner et al., 2022), to ‘allochthonous’ minority, non-dominant, or migrant groups (Gruner et al., 2022; Jie et al., 2022; Klenner et al., 2022; Le et al., 2022; Leyerzapf & Abma, 2017; Pimentel et al., 2021; Sawatsky et al., 2017; Shepherd, 2019), to refugees or people born outside their country of residence (Gruner et al., 2022; Rothlind et al., 2021; Sorensen et al., 2019), or to race and ethnicity (Campinha-Bacote, 2002; Hordijk et al., 2019; Jie et al., 2022; Leyerzapf & Abma, 2017; Liu & Li, 2022; Matthews & Van Wyk, 2018; Pang et al., 2021; Sklar, 2018; Uzelli Yilmaz et al., 2022; Vasquez Guzman et al., 2021). These characteristics, moreover, were presented as static facts. This interpretation mainly found expression in the cultural competence (Box 1) and cultural safety approaches.

The implication of this interpretation for education, as pointed out in the analysed articles, was that both students and teachers should undergo specific skills-oriented training to acquire cultural knowledge, skills, attitudes, and awareness (Brottman et al., 2020; Campinha-Bacote, 2002; Gruner et al., 2022; Guerra & Kurtz, 2017; Hordijk et al., 2019; Klenner et al., 2022; Kor et al., 2022; Leyerzapf & Abma, 2017; Matthews & Van Wyk, 2018; Pang et al., 2021; Pimentel et al., 2021; Sorensen et al., 2019; Vasquez Guzman et al., 2021) about the values, habits, and traditions of specific categories of patients (Ang et al., 2020; Brottman et al., 2020; Hordijk et al., 2019; Jowsey, 2019; Le et al., 2022; Liu & Li, 2022; Nazar et al., 2015; Sorensen et al., 2019). It was also suggested to organise more interactions with people of the specific group during rotations or (online) travels (Kor et al., 2022; Pang et al., 2021), via community projects (Gruner et al., 2022; Vasquez Guzman et al., 2021), as standardized simulation patients (Uzelli Yilmaz et al., 2022), or virtual patients (Rothlind et al., 2021). Due to the busy nature and demands of clinical work, this approach was found to be more common during clinical rotations than during classroom-based education (Nazar et al., 2015), but cultural competence development in clinical placements was also encouraged through immersion, interaction, observation, and reflection (Liu & Li, 2022).

#### Critical views

However, in several of the 52 articles under scrutiny, this interpretation of ‘culture’ was challenged, with some authors emphasising that ‘there is more variation within ethnic groups than across ethnic groups (intra-ethnic variation)’ (Campinha-Bacote, 2002), ‘there is often overlap between other vulnerable populations’ (Gruner et al., 2022), and that discussions about culture stimulate ‘a variety of strong emotional triggers for students’ (Worden & Ait-Daoud Tiouririne, 2018). Other authors went on to argue that this conceptualisation was overly simplistic (Jie et al., 2022; Jowsey, 2019), prompted othering and lead to culture being seen as a problem (Jie et al., 2022), overlooked intracultural diversity and structural factors (Le et al., 2022; Vasquez Guzman et al., 2021), had the potential to reinforce stereotypes (Ang et al., 2020; Sawatsky et al., 2017; Solchanyk et al., 2021), and that ‘information about cultures [could not] … be reduced to a defined point in time and location’ (Clementz et al., 2017), nor to ‘lists of attributes … that exoticize patients’ (Dao et al., 2017). Finally, researchers objected that the term ‘culturally competent’ could be taken to suggest that ‘there is a theoretically finite body of knowledge that can be mastered to become culturally competent’ (Brottman et al., 2020), which was coined an ‘unattainable goal’ (Martinez et al., 2015). In summary, these critical voices warned that if we regard culture as a fixed patient characteristic, we risk simplifying patients, frustrating students, and promoting unrealistic learning.

### Culture as multiple fixed characteristics

We found that some other approaches recognised that not only patients, but also students and doctors have multiple co-existing identities (including race, socioeconomic class, gender, sexual orientation, language proficiency, and cognitive ability), thereby addressing the aforementioned limitations of focusing on fixed characteristics of a specific category of patients (Blackie et al., 2019; Bochatay et al., 2022; Eckstrand et al., 2016; Muntinga et al., 2016; Peek et al., 2020; Shepherd, 2019; Sklar, 2018; Uzelli Yilmaz et al., 2022). Advocates of the respective approaches implicitly or explicitly considered culture as one such form of identity that influences other identities. Keen to recognise that cultural diversity can have different meanings, Muntinga et al. (2016) distinguished between a ‘narrow’ and ‘broad’ view on culture, with the former encompassing characteristics such as ethnicity, nationality, language, and migrant status and the latter expanding this interpretation to also include other biological, social, and cultural categories, such as gender and class. This broad notion is mainly manifested in the intersectionality approach that is based on the assumption that multiple identities ‘intersect’ (Box 2).

**Box 2:** Description of Intersectionality, identified in the articles included in the critical review on the meanings of cultural diversity (2015–2022)

**Intersectionality**: ‘holds that we are all made up of many different intersecting identities’ (Blackie et al., 2019). It is ‘a critical strand of diversity theory that assumes that people occupy unique social locations based on multiple coexisting and mutually reinforcing social identities (such as gender, sexuality, social class, race and ethnicity)’ (Muntinga et al., 2016)	

As practical implication of this notion for medical education it was reported that case descriptions and pictures should include categories of differences other than culture, sex/gender, and class as equally important patient identities (Eckstrand et al., 2016; Muntinga et al., 2016). Authors of several articles furthermore suggested that doctors should be encouraged to reflect on their social class and immigration status, age, sexuality, gender, and geographic location (Hordijk et al., 2019).

#### Critical views

Yet, we again found that some authors of the analysed articles criticised this notion of culture as multiple fixed characteristics. More specifically, they argued that although it recognises people’s multiple interrelated characteristics (of oneself and of the other), they are still constructed as static, thereby disregarding the possibility that varying and altering understandings, contexts, and relations play a role. In the words of Muntinga et al. (2016), such approach could ‘increase the risk of simplifying the complexity of lived experiences, and …. should always be used cautiously in order to prevent the homogenisation of patient experiences and bodies’. In the following, we will elucidate this more elaborate conceptualisation of culture.

### Culture as a dynamic outcome impacting social interactions

In the third notion we identified, cultural diversity was conceptualised as the product of interactions that were constantly changing and, rather than being pertinent to specific groups as fixed identities only, were relevant for *all* people. More specifically, culture was explicitly and implicitly defined as ‘a complex, dynamic phenomenon which evolves over time’ (Clementz et al., 2017; Solchanyk et al., 2021) and in terms of socially transmitted patterns of shared meanings and human behaviour including attitudes, thoughts, world views, communications, ideas, values, norms, actions, traditions, customs, and beliefs (Brottman et al., 2020; Dogra et al., 2016; Hordijk et al., 2019; Jie et al., 2022; Jowsey, 2019; Matthews & Van Wyk, 2018; Shepherd, 2019; Sklar, 2018; Solchanyk et al., 2021). More explicitly than in the previous notion, adherents of this view accepted that patients, their relatives, and health professionals each have their own culture that may impact doctor-patient interaction (Dogra et al., 2016; Klenner et al., 2022; Martinez et al., 2015). That is to say, they posited that cultural differences impact how doctors, patients, students, and teachers interact. Examples of such differences were individualistic values that conflicted with collectivist beliefs (Shepherd, 2019) or dissimilarities between the culture of medicine (Kuper et al., 2016; Manca et al., 2020) and that of the non-medical world: ‘health care environments, including educational ones, also have cultures that influence and shape the actions of practitioners and patients alike’ (Jowsey, 2019). As such, culture was constructed as both an outcome and influencer of interactions, and thus dynamic. This interpretation became manifest in several approaches, but in the cultural humility approach especially, which aims to cultivate a mutually respectful patient-physician relationship (Box 3).

**Box 3:** Description of Cultural humility, identified in the articles included in the critical review on the meanings of cultural diversity (2015–2022)

**Cultural humility**: ‘facilitates awareness of one’s own and others’ cultural beliefs and practices’ and encourages patient-focused care (Nazar et al., 2015). As such, it is valued for its lifelong learning process and commitment (Brottman et al., 2020; Campinha-Bacote, 2002; Eckstrand et al., 2016; Martinez et al., 2015), aimed at self-reflection and self-evaluation (Eckstrand et al., 2016; Martinez et al., 2015; Nazar et al., 2015). Cultural humility emphasizes curiosity about a patient as expert of their own experience. It aims to cultivate a mutually respectful patient-physician relationship, and it is a dynamic process with social justice goals at the core (Solchanyk et al., 2021)	

We found that this interpretation of culture, too, carried implications for educational practice in terms of curriculum contents and design. One author, for instance, reported that an intervention at the University of Antwerp was transformed from a simulation training that focused on learning facts about migrants into one that offered a range of diversity awareness experiential exercises with simulated patients from diverse backgrounds (Ang et al., 2020). Rather than focusing on patients, courses that embrace this notion of culture promote reflection on and discussion of students’ own assumptions, biases, and perspectives (Brottman et al., 2020; Clementz et al., 2017; Jie et al., 2022; Jowsey, 2019; Martinez et al., 2015), preferably through (online) small-group discussions (Brottman et al., 2020; Martinez et al., 2015; Solchanyk et al., 2021) rather than in lecture format only (Brottman et al., 2020; Nazar et al., 2015). Another suggestion is to involve students in curriculum review and design activities (Krishnan et al., 2019; Martinez et al., 2015), since their ‘lived experiences … are valued as educational resources when learning about diversity’ (Nazar et al., 2015). Also community members should be involved in curriculum design (Fung & Ying, 2022; Goez et al., 2020) so as to ensure that curriculum learning objectives and contents incorporate attention to patients’ lived experiences and ‘synthesize the biological pathways of disease with environmental phenomenon’ (Martinez et al., 2015). Other examples mentioned are bi-directional student exchange, collaboration with social workers, and courses in humanities, which stimulate self-reflection and open mindedness, also for educators (Solchanyk et al., 2021).

#### Critical views

This third conceptualisation of culture was disputed by some authors who argued that cultural differences and interactions between the stakeholders involved, rather than being neutral, are characterised by impactful hierarchical relations. As such, this perception disregarded the fact that power differentials are inherent in health services delivery and safety (Guerra & Kurtz, 2017). Exponents of such a view therefore argued that, although regarding culture as dynamic and all-encompassing was a plus, it overlooked how certain cultural views are unconsciously valued more and have a higher status than others, and how this integrated bias impacts the whole health care and educational system.

### Culture as power dynamics

In response to the aforementioned critiques, some researchers argued that cultural diversity should be conceptualised in terms of a professional field where differences are characterised by power dynamics. Blackie et al. (2019), for instance, suggested that systems of inequality and power imbalances are related to people’s multiple and mutually reinforcing identities (Blackie et al., 2019; Eckstrand et al., 2016; Peek et al., 2020). To demonstrate their case, authors pointed to the inherent inequality of all sorts of differences, including: patient-physician relations (Brottman et al., 2020; Eckstrand et al., 2016; Guerra & Kurtz, 2017; Jie et al., 2022; Martinez et al., 2015; Nazar et al., 2015), educators’ norms that conflict with those of their learners (Manca et al., 2020), the advantage of white learners’ access to the language of medicine before entering the medical community compared to minoritized learners who did not have this access (Zaidi et al., 2021a, 2021b), educators’ silence on racial issues in curricular content (Zaidi et al., 2021a, 2021b), ‘normal ranges’ that are primarily based on Caucasian populations (Sharma & Kuper, 2017), and ‘medicine’s traditional dominance over other professions’ and ‘its ongoing legacy of use as an agent of colonisation’ (Kuper et al., 2016). These unconscious and integral inequities and power structures were argued to impact pedagogies, the organisation’s structure and procedures, clinical interactions, and curriculum contents. Authors furthermore contended that the biomedical model is based, albeit unconsciously, on Western perspectives (Martinez et al., 2015; Matthews & Van Wyk, 2018) and on the assumption that ‘white is the default race when none is mentioned’ (Sharma & Kuper, 2017). This conceptualisation was espoused by the approaches of DEI/EDI (diversity, equity, inclusivity), social accountability, intersectionality, cultural safety, cultural humility, race consciousness, the Foucauldian perspective, and especially by the critical consciousness approach (Box 4).

**Box 4:** Description of Critical consciousness, identified in the articles included in the critical review on the meanings of cultural diversity (2015–2022)

**Critical consciousness**: is ‘a state of understanding how power and difference shape social structure and interaction (“reading the world”)’ (Dao et al., 2017), ‘a “reflective awareness” of power, privilege and inequities lodged within social relationships’ (Nazar et al., 2015; Zaidi et al., 2016, 2017) that ‘has been conceptualised in medical education as an intellectual construct to foster a reflexive awareness of professional power in health care, to unearth the values and biases legitimizing medicine as currently practiced, and to foster transformation and social accountability’ (Manca et al., 2020)	

As one of the practical implications authors pointed to the importance of creating a safe space (Herzog et al., 2021; Holdren et al., 2022; Jie et al., 2022; Zaidi et al., 2017) and ‘being facile to pain’ so as to encourage cultural discussion and create an understanding about power and privilege in society (Zaidi et al., 2016, 2017). Another suggestion was to ask students ‘to complicate their relationships to biomedical knowledge, reconsider hierarchical dynamics in the clinic, and re-centre the struggle against social causes of illness at the heart of their future practices of medicine and medical research’ (Cavanagh et al., 2019). Manca et al. (2020), moreover, noted that ‘medical educators should help learners identify the values, assumptions, and sociopolitical forces shaping the structure of health care and practice and also provide opportunities to question them’. Zaidi et al., 2021a, 2021b suggested students and staff should be empowered to be able to resist racist structures rather than conform to the current system, and also Gruner et al. (2022) mentioned enhancement of students’ advocacy skills. Participatory co-design of institutional policies, engaging individuals who experience discrimination and harassment themselves, was another suggestion (Sukhera et al., 2022). One way to implement critical consciousness in practice is by applying the Introduction to Medicine and Society (IMS) model which treats the concept ‘as the physician’s always-evolving orientation to navigating relationships in internal, interpersonal, and structural domains’ (Dao et al., 2017). Finally, authors proposed that students be offered history lessons on racism, discrimination and disparities (Guerra & Kurtz, 2017; Martinez et al., 2015), and discuss humans’ shared origins (Clementz et al., 2017) so that they will be encouraged ‘to critically examine the construct of race and ethnicity as a proxy variable in complex disease outcomes’ (Martinez et al., 2015).

#### Critical views

Our analysis, however, again revealed that this broad, system-focused interpretation of culture was met with scepticism, although in comparison with the critical views on the previous notions, this scepticism was more concerned with the operationalization rather than the conceptualization of the notion. Some authors of the articles we reviewed warned that it could be challenging to implement the critical consciousness approach (Halman et al., 2017; Manca et al., 2020). For instance, it would be difficult for white people to recognise and relinquish their privileged position (Jowsey, 2019), not to mention that the theoretical underpinnings of critical consciousness are at odds with the dominant competency-based and biomedical knowledge construction approaches in medical education and research (Halman et al., 2017; Manca et al., 2020; Zaidi et al., 2021a, 2021b). Existing discrimination and harassment policies would allow for power to be maintained and controlled by those in power (Cavanagh et al., 2019). ‘Major structural and cultural transformations within medical education’ (Manca et al., 2020) would be needed to address these challenges, as well as a ‘careful, reflexive attention to language and performance in clinical and educational contexts' (Bleakley, 2017). Only a system-based approach focusing on the intersection of diversity, equity, and inclusivity could ‘reach the ultimate goal of creating an environment that promotes belongingness for everyone’ (Rossi et al., 2022). Finally, authors noted that awareness of existing hierarchical relations does not eliminate the bias that is deeply ingrained in medical education, research, and health care systems, nor does it obviate the difficulty for an individual teacher or doctor, or even a single organisation or school to tackle it.

## Discussion

In medical education, several approaches to cultural diversity exist that each may espouse a different conceptualisation of ‘cultural diversity’. With this study, we sought to unpack these various conceptualisations, comparing approaches, and showing their relative practical strengths and limitations in the process. In doing so, we complemented earlier reviews that focused on one approach only (such as critical consciousness or cultural safety). Adding to these earlier studies, our comparison showed how the notion of cultural diversity not only challenges people’s mind-sets and communication skills, it also touches upon more fundamental aspects of medical practice and knowledge construction.

We identified four different interpretations of ‘cultural diversity’. First, we showed that some approaches upheld the notion of ‘fixed patient characteristic’, conceptualising cultural differences in terms of one static characteristic. Arguing that such perception risked simplifying patients and frustrating students, some researchers advocated a second notion of ‘multiple fixed characteristics’ that did recognise the fact that people have multiple interrelated characteristics. As they disregarded the possibility that changing understandings and contexts play a role, a third notion came into view that accepted cultural diversity as a ‘dynamic outcome impacting social interactions’. This dynamic and all-encompassing concept, however, did not factor in how hierarchical relations impact interactions. Exponents of the fourth notion therefore viewed cultural diversity as the product of ‘power dynamics’, where differences are characterised by inequalities. The above notions all influence perceptions of how students should be taught about patient variances, in turn impacting educational contents and design. The consequences of the fourth notion, however, are more fundamental, for they affect theories and principles underlying medical education, research and practice.

In line with our critical theory paradigm we looked into these underlying power structures, and ontological and epistemological consequences. The knowledge that biomedical research and knowledge construction are permeated by inherent inequalities is not new. It is increasingly recognised that basing research evidence on standardised populations in clinical trials, which historically include white middle-aged males only, leads to both an overestimation and an underestimation of medically relevant social and biological variety between sexes, ages, and ethnicities (Keville, 1994; Lock & Nguyen, 2018; Nordell, 2021; Perez, 2019). To actually change these practices and include more variety in clinical trials, however, is no easy feat without practical precedents (Salive, 2017; Saturni et al., 2014; Stronks et al., 2013; Taipale et al., 2022). In addition to this, the limitations to the different notions of ‘cultural diversity’ we previously identified apply equally to medical knowledge construction: by merely including more categories in clinical research, the complexity of people’s lived experiences and the impact of hierarchical relations on interactions is still disregarded. That is to say, patients’ medical issues always ‘happen’ in relation to their biological and social environment and their position in society matters, also in the doctor’s office. We therefore suggest to integrate patients’ lived experiences and relationships into medical research, so that not only the biomedical processes, but also the contexts in which they occur and their concomitant (hierarchical) interactions are considered, as also argued by Shim (2005) and Wyatt et al. (2022). We recommend that researchers employ methodologies that look beyond fixed categories. These research methodologies would also satisfy opponents of evidence-based medicine (EBM) (Felder and Meerding, 2017; Greenhalgh et al., 2014) such as Felder and Meerding (2017). They noted that ‘chances are slim that an individual patient complies with the population of an RCT [randomised controlled trial]’ and that ‘EBM pictures disease and treatment as too abstract, simplified and objectified, which does not do justice to the patient’s pluralistic world’ (Felder and Meerding, 2017). However complex, it is essential that we change these research methodologies so that medical research becomes more relevant for medical treatment.

Yet, inequalities not only exist on a methodological level, impacting how knowledge is constructed; they can also be found on an epistemological and ontological level: in the knowledge itself. Although biomedical science is purportedly neutral and universal, it embraces paradigms that are based on Western notions of, for example, the body-mind dualism, individualism, and biological reductionism (Lock & Gordon, 2012; Wong et al., 2021). What is generally understood to be ‘objective’ scientific knowledge within medicine and beyond, in effect represents a very small proportion of the global knowledge capital (Hall & Tandon, 2017). The harmful implications of inequalities are considerable, which the Tuskegee Study, a historical and well-known example, illustrates (CDC, 2023).

Hence, to maximise the benefit to all patients around the world, it is imperative that we raise awareness of these hierarchies in science and decolonise knowledge (Hall & Tandon, 2017).

As previously noted in the Results section, these insights carry fundamental implications for education that are laborious and difficult to implement. Moreover, it seems that the identified approaches and their practical implications themselves are often tailored towards a privileged audience. For example, when critical consciousness is framed to ‘increase awareness of hierarchical relations’, it is assumed that the people involved are not aware in the first place, whereas many students, teachers, and patients have immense knowledge, skills and experience about inequalities. As a start, however, and taking these assumptions into account, the following suggestions that also make reference to other paradigms and policies in health care and medical education might be worth considering. Consistent with both the intersectionality approach and the person- or patient-centred health care paradigm, we propose that curricula standardly include information on patients’ multiple identities and lived experiences in case descriptions and that they zoom in on the needs of the individual patient (Kitson et al., 2013; Stichting-mens-achter-de-patient, 2022; Wang, 2021). Moreover, we suggest that curricula take the cultural humility approach as an example by encouraging teachers and students to share their own experiences with unequal treatment, to reflect more on their own values and hierarchical position and how these impact the doctor-patient and student–teacher relation, and by recognising that such reflection is crucial to develop a professional identity (Sawatsky et al., 2017; Wald, 2015). Lastly, we recommend that curricula draw inspiration from the critical consciousness approach and pay more attention to the impact of power imbalances. This strategy would entail teaching students more explicitly about the power of medical knowledge, making use of the existing daily experiences of underrepresented students and teachers, to question ‘what is medical research evidence?’, and how to use this evidence in clinical reasoning. Aiming to decolonise medical curriculum policies, such new take on cultural diversity teaching would also require educators to acknowledge Western epistemological hegemony, to explicitly include literature from diverse sources, and to monitor diversity-integrated topics in the curriculum (Heleta, 2016; Wong et al., 2021). The IMS model previously mentioned, but also the AMEE Guide (Dogra et al., 2016), Martinez’s 12 tips (Martinez et al., 2015), and experiences at UCL Medical School (Wong et al., 2021) are just a few examples of educational initiatives already taken to implement this broad and integrated approach. A first step in effecting the fundamental system change so needed is to collect and review more of these examples and to conduct additional reflective research on the medical system.

However, it is crucial that such future research explicitly include non-Western authors and journals. It is true that for this study we selected the eight top journals in the field reasoning that these journals together reach the widest readership and reflect state of the art approaches and conversations. These journals, however, are predominantly Western-focused, dominated by scholars from high-income countries, and written by and for a privileged audience. We realize that this article is in fact no exception. Our selection of journals does not reflect the long history that many of the discussed approaches have, most of which has been shaped by Black, Indigenous and Eastern scholars, outside of health professions education, such as K. Crenshaw (intersectionality) (Crenshaw, 2017), P. Freire (critical consciousness) (Freire, 2021), and M. Tervalon and J. Murray-Garcia (cultural humility) (Tervalon & Murray-Garcia, 1998). In hindsight, we should also have included non-dominant literature as sources of inspiration. As suggested in one non-Western-focused article (Matthews & Van Wyk, 2018), our selection probably led to a biased analysis of a Western and privileged approach to ‘cultural diversity’. In order to limit such bias and contribute to the decolonisation of medical research and health professions education research, we invite future researchers to specifically look for publications in journals that are based in a diversity of regions globally, and adapt their research methodologies and distribution practices to include non-Western and underprivileged participants and viewpoints (Ajjawi et al., 2022; Hall & Tandon, 2017; Louw, 2019). Such future research could also look into the role history and context play in the conceptualisation of ‘cultural diversity’.

## Conclusion

Although the main aim of cultural diversity training is to reduce health inequalities, it seems that the medical system itself is riddled with inequalities, requiring a fundamental transformation of medical practice and biomedical knowledge construction. If we want to teach students how to treat a diverse patient population, we must first acknowledge that: 1) medical issues always ‘happen’ in relation to their biological and social environment, 2) patient variety is the norm, not an exception, and that 3) biomedical knowledge is not neutral. It will take time and effort to integrate these principles into research and educational practice. Yet, educators can learn from first initiatives and consider the practical suggestions offered in this study. Awareness of the implicit meaning of and the consequences of referring to ‘cultural diversity’, is paramount.
